# Metabolome and Transcriptome Analyses of Anthocyanin Accumulation Mechanisms Reveal Metabolite Variations and Key Candidate Genes Involved in the Pigmentation of *Prunus tomentosa* Thunb. Cherry Fruit

**DOI:** 10.3389/fpls.2022.938908

**Published:** 2022-06-29

**Authors:** Aidi Zhang, Haiying Yang, Shujun Ji, Changping Tian, Ni Chen, Hansheng Gong, Jianzhao Li

**Affiliations:** ^1^School of Food Engineering, Ludong University, Yantai, China; ^2^Cherry Research Department, Yantai Agricultural Science and Technology Institute, Yantai, China; ^3^Fushan Agricultural Technology Extension Center, Yantai, China; ^4^The Engineering Research Institute of Agriculture and Forestry, Ludong University, Yantai, China

**Keywords:** Tomentosa cherry, fruit, anthocyanin, metabolome, transcriptome

## Abstract

*Prunus tomentosa* Thunb. has excellent nutritional, economic, and ornamental values with different fruit color. The red coloration of fruit is determined by anthocyanin pigmentation, which is an attractive trait for consumers. However, the mechanisms underlying fruit color formation in the *P. tomentosa* cherry are not well understood. In this research, the pigmentation patterns in red-color *P. tomentosa* (RP) fruit and white-color *P. tomentosa* (WP) were evaluated. Anthocyanin content in matured RP fruit was significantly abundant compared with WP fruit. Metabolomic profiling revealed that pelargonidin 3-*O*-glucoside, cyanidin 3-*O*-rutinoside, and pelargonidin 3-*O*-rutinoside were the predominant anthocyanin compounds in the RP fruit, while, WP fruit had less anthocyanin compositions and lower level. Then, integrative analyses of transcriptome and metabolome identified 285 significant differentially expressed genes (DEGs) closely related to anthocyanin differentially expressed metabolites (DEMs). Among them, nine genes were involved in anthocyanin biosynthesis, transport and degradation pathway, including four biosynthesis genes (*PtPAL1*, *PtDFR*, *PtANS, and PtUFGT*), two transport genes (*PtGST11*, *PtABC10*), and three degradation genes (*PtPOD1*, *PtPOD16, PtPOD73*). Transcriptome data and real-time PCR showed that the transcript levels of biosynthesis and transport genes were significantly higher in RP than in WP, especially *PtANS, PtUFGT,* and *PtGST11*, suggesting they may play key roles in red-colored fruit formation. Meanwhile, the degradation-related genes *PtPOD1/16/73* took on exactly opposite trend, suggesting their potential effects on anthocyanin degradation. These results provide novel insights into color patterns formation mechanisms of cherries fruit, and the candidate key genes identified in anthocyanin biosynthesis, transport and degradation may provide a valuable resource for cherry breeding research in future.

## Introduction

Anthocyanins are a group of important natural water-soluble pigments, belonging to the flavonoid family as plant secondary metabolites, which are widely distributed in nature (Holton and Cornish, 1995; Tanaka and Ohmiya, 2008; Jaakola, 2013). In plants, anthocyanins have various biological and physiological functions, including facilitating pollination and seed transmission, endowing plants with biological and abiotic stress resistance, and prolonging fruit life (Jaakola, 2013; Zhang et al., 2013). The effects of anthocyanins on human health have also been studied, as they have shown health-promoting properties, including anti-oxidative, anti-microbial, anti-diabetic, anti-cancer, anti-inflammatory, anti-obesity, and cardiovascular disease preventive effects, and thus may serve as potential pharmaceutical ingredients (Holton and Cornish, 1995; He and Giusti, 2010). Thus, anthocyanin-rich foods, with attractive colors and suggested benefits, have been increasingly studied and have become popular among the general public (Pojer et al., 2013). In nature, anthocyanins exist ubiquitously as glycosylated polyphenolic compounds (Tanaka and Ohmiya, 2008), mainly including cyanidin, pelargonidin, delphinidin, peonidin, petunidin, and malvidin, which are responsible for red, orange, and purple to blue colors (He and Giusti, 2010; Jaakola, 2013).

Anthocyanin accumulation in plants depends on the balance between biosynthesis, transport, and degradation (Holton and Cornish, 1995; Passeri et al., 2016). Compared with transport and degradation, anthocyanin biosynthetic pathways have been extensively studied in many plant species, such as tomato (Goldsbrough et al., 1994), apple (Kondo et al., 2002), pear (Wang et al., 2017), grape (Kobayashi et al., 2001, 2004), kiwifruit (Li et al., 2018; Wang et al., 2022), olive (Iaria et al., 2016; Carbone et al., 2019), and sweet cherries (Liu et al., 2013; Shen et al., 2014). Anthocyanins are synthesized from the precursor substrate phenylalanine through a series of structural genes encoding enzymes (Koes et al., 2005; Jaakola, 2013; Iaria et al., 2016). The phenylalanine is successively converted to cinnamic acid, 4-coumaric acid, and 4-coumaroyl-CoA, catalyzed by phenylalanine ammonia-lyase (PAL), cinnamate-4-hydroxylase (C4H), and 4-coumarate-CoA ligase (4CL), respectively. Then, 4-coumaroyl-CoA is transformed into dihydroflavonols (precursors of flavonols and anthocyanins) by early biosynthesis genes (EBGs), including chalcone synthase (CHS), chalcone isomerase (CHI), and flavanone 3-hydroxylase (F3H). The late biosynthetic genes (LBGs) involved in the late anthocyanin biosynthesis pathway are dihydroflavonol 4-reductase (DFR), anthocyanidin synthase/leucoanthocyanidin dioxygenase (ANS/LDOX), and UDP-glucose: flavonoid-3-*O*-glucosyltransferase (UFGT). The LBGs acting as downstream genes in anthocyanin biosynthesis pathways have a specific role in anthocyanin biosynthesis (Lepiniec et al., 2006; Guo et al., 2014). Positive correlations between anthocyanin content and expression levels of LBGs have been consistently observed in plants, such as *BrDFR8*/*9* in *Brassica rapa* L. (Ahmed et al., 2014), *AaLDOX* in kiwifruit (Li et al., 2018), *ZjANS* and *ZjUGT79B1* in Chinese jujube (Shi et al., 2020), and *pUFGluT* in apple (Kondo et al., 2002). Additionally, *DFR* has been reported as a visual, non-destructive, and plant-derived marker gene for tomato anthocyanin pigmentation (Goldsbrough et al., 1994).

After being synthesized in the cytoplasmic face of the endoplasmic reticulum, the anthocyanins are transported to the vacuole for storage by glutathione S-transferase (GST), ATP-binding cassette (ABC), and multi-drug and toxic compound extrusion (MATE) families (Marrs et al., 1995; Goodman et al., 2004; Gomez et al., 2009). Among them, the effects of *GSTs* on anthocyanin transport have been widely recognized. *TRANSPARENT TESTA 19* (*TT19*, a GST gene) in *Arabidopsis* has been demonstrated to be necessary for the transport of anthocyanins into vacuoles (Kitamura et al., 2004). *RAP* (*reduced anthocyanins in petioles*), which encodes GST, has been shown to affect the fruit color of strawberries, as the principal transporter for anthocyanins (Luo et al., 2018). In apple, *MdGSTF6* has been demonstrated to act as an anthocyanin transporter, thus affecting anthocyanin accumulation (Jiang et al., 2019). *PpGST1* plays an indispensable role in peach fruit coloration, as it not only regulates anthocyanin accumulation, but also affects the expression of anthocyanin biosynthetic and regulatory genes (Zhao et al., 2020). To date, many reports have indicated that anthocyanin accumulation is also associated with anthocyanin degradation. In strawberries, anthocyanin degradation is mainly catalyzed by POD (peroxidases) and LAC (laccases), which may be closely related to *POD3*, *POD6*, *POD63*, *laccase 9*, and *laccase 14* (Zhang et al., 2019). One LAC-predicted coding gene and two POD-predicted coding genes have been identified in anthocyanin degradation in “Red Bartlett” pears (Wang et al., 2017). In *Brunfelsia calycina* Benth. flowers, *BcPrx01*, a basic vacuolar peroxidase, has been shown to be responsible for anthocyanin degradation (Zipor et al., 2015). In apple, the transcription level of *MpPOD1*/*8*/*9* was upregulated together with anthocyanin degradation in high-temperature treatment fruits (Rehman et al., 2017).

*Prunus tomentosa* Thunb. is part of the *Prunus* subgenus *Lithocerasus*, along with other cherries, belonging to the Rosaceae family (Zhang et al., 2008; Hamilton et al., 2016). *P. tomentosa* is also known as Tomentosa cherry or Nanking cherry, which is native to northern China and has been naturalized into Russia, Japan, and other northern regions as an important deciduous fruit tree with high economic and ornamental values (Zhang et al., 2008; Hamilton et al., 2016). In addition to its diverse adaptability to various environments, the Tomentosa cherry has not only been widely used as rootstock for sweet cherry, peach, and plum, but it is also an excellent gene donor for improving *Prunus* species (Zhang et al., 2008; Zhang and Gu, 2016). *P. tomentosa* seeds, as by-products, have anti-inflammatory and anti-oxidant activities, causing them to be highly regarded (Liu et al., 2014). Moreover, the fruits of *P. tomentosa* possess many valuable traits, including their unique taste, various colors, and rich accumulation of vitamins and antioxidants (Cao et al., 2015; Hamilton et al., 2016). Although *P. tomentosa* cherry fruits can be eaten fresh, they are more suitable for making preserves, jam, fruit juices, and beneficial extracts (Hamilton et al., 2016).

In these fruits, the red color of the fruit flesh and peel is mainly due to the accumulation of anthocyanins, however, the composition and content of specific anthocyanins vary with different cherry varieties (Blando and Oomah, 2019). Cao et al. (2015) have evaluated seven anthocyanins in four cherry species (*Prunus avium* L., *Prunus cerasus* L., *Prunus pseudocerasus* Lindl., and *P. tomentosa*) by HPLC-ESI-MS/MS analysis, and found that cyanidin 3-rutinoside was predominant in *P. avium*; and cyanidin 3-glucosilrutinoside and cyanidin 3-rutinoside were chief components in both *P. cerasus* and *P. pseudocerasus*. Meanwhile, red *P. tomentosa* had a different anthocyanin profile, where pelarogonidin-3-rutinoside was the dominant compound. Moreover, cyanidin 3-*O*-glucoside and cyanidin 3-*O*-rutinoside were the major anthocyanins in sweet cherry (*P. avium*), sour cherry (*P. cerasus*) and black cherry (*Prunus serotina* Ehrh.; Homoki et al., 2016; Acero et al., 2019; Brozdowski et al., 2021). In anthocyanin biosynthesis pathways, six biosynthetic anthocyanin genes (*PacCHS*, *PacCHI*, *PacF3H*, *PacDFR*, *PacANS*, and *PacUFGT*) have been significantly correlated with anthocyanin synthesis in a red-colored cultivar (“Hongdeng”), while only *PacUFGT* appeared to be significantly correlated with anthocyanin synthesis in a bi-colored cultivar (“Caihong”; Liu et al., 2013). Moreover, the expression levels of the late genes (*PacDFR*, *PacANS*, and *PacUFGT*) were in accordance with the pattern of anthocyanin accumulation during “Hongdeng” fruit development (Shen et al., 2014). A study based on four cherry cultivars has identified eight anthocyanin biosynthesis genes (*PaPAL*, *PaCHS*, *PaCHI*, *PaF3H*, *PaF3’H*, *PaDFR*, *PaANS*, and *PaUFGT*; Starkevič et al., 2015). In addition, *PavMYB10.1* formed a putative MBW complex with *PavbHLH* and *PavWD40*, then binding to the *PavANS* and *PavUFGT* promoter to improve anthocyanin accumulation (Jin et al., 2016). Recently, post-harvest UV-C treatment effectively regulated flavonoid and anthocyanin synthesis, as well as increasing the expression of *PAL*, *4CL*, *C4H*, *CHS*, *CHI*, *F3H*, *DFR*, *ANS*, and *UFGT* genes in the “Brooks” cultivar (Zhang et al., 2021). Our previous work in “Hongdeng” revealed that *PacANS* exhibited the highest transcript level, with respect to anthocyanin content, through RNA-seq and WGCNA (Yang et al., 2021a).

Integrated transcriptomics and metabolomics technologies have been widely used to investigate differentially expressed genes (DEGs) and to reveal the biosynthesis pathways and regulators of metabolites in plants, such as *Arabidopsis thaliana* (Masclaux-Daubresse et al., 2014), tomato (Enfissi et al., 2010), jujube fruit (Shi et al., 2020), and so on. In this study, we aimed to analyze the color difference between red and white *P. tomentosa* cherries, identify anthocyanin metabolites, and recognize the DEGs involved in anthocyanin biosynthesis, transport, and degradation. The anthocyanin components in *P. tomentosa* cherries were identified through LC–MS/MS analysis, and insights into the color change of the fruit were obtained through integrated metabolomics and transcriptomics analysis. Our results provide important insights for the identification of functional genes and metabolites involved in anthocyanin formation and color change in red and white *P. tomentosa* fruits, laying a molecular foundation for color improvement and the creation of new fruit colors through the use of advanced breeding technologies.

## Materials and Methods

### Plant Materials

Fruits of *P. tomentosa* cherry were used as plant materials in this work, which were collected from the orchard of Yantai Agricultural Science and Technology Institute, Shandong Province, China (latitude: 37.4893, longitude: 121.2790; elevation: 10.2 m), in 2019. The red-color *P. tomentosa* (RP) and white-color *P. tomentosa* (WP) fruits grow on different trees. And the fruits with red or white color were harvested from three trees, respectively, at mature stage (45 days after full bloom), and healthy cherry fruits with uniform size and no defects were selected for the experiments. Fruit materials (mixture of peel and flesh) were collected, frozen in liquid nitrogen, and stored at −80°C for subsequent analyses. All experiments were performed with three biological replicates, each replicate consisting of 10 fruits.

### Measurements of Fruit Firmness and Color

Measurements of cherry fruit firmness were performed using a CT310K texture analyzer (AMETEK Brookfield, Middleboro, MA, United States) equipped with a cylinder probe (2 mm diameter). The analysis conditions of the probe were as follows: Press distance, 3.0 mm; press speed, 2.3 mm/s; trigger force, 0.05 N. Firmness is expressed as peak force (N). The color of cherry fruits was monitored using a HunterLab chromameter (Konica Minolta, Inc., Japan) with the CIE L^*^a^*^b^*^ color space. Measurements were performed on two different flat surfaces of each cherry fruit. Each sample point had three biological replicates, with 10 cherries per replicate.

### Determination of the Total Anthocyanin Content

Extraction and measurement of the total anthocyanin content was carried out employing the HCL–methanol method described previously by Yang et al. (2021a) Two buffer systems were employed in this experiment, one with 0.4 M potassium chloride buffer (pH 1.0), and the other with 0.4 M dibasic sodium phosphate buffer (pH 4.5). Approximately 0.5 g of cherry fruits ground with liquid nitrogen was soaked in 2.5 ml of 0.01% (v/v) HCL–methanol solution and extracted at 24°C in the dark for 24 h. After centrifugation at 12,000 rpm for 20 min, a UV-723 N spectrophotometer (Youke, Shanghai, China) was applied to measure the absorbance of the solutions at 510 and 700 nm in buffers at pH 1.0 and 4.5. The anthocyanin content was calculated using the following equation: A = [(A510 − A700) _pH 1.0_ − (A510 − A700) _pH 4.5_], and converted into mg cyanidin 3-galactoside per 100 g fresh weight (FW). Three independent biological replicates for each point were analyzed.

### Metabolomic Profiling Analysis

The freeze-dried cherry samples were crushed to powder using a mixer mill (MM 400, Retsch) for 1.5 min at 30 Hz, which was equipped with zirconia beads. Briefly, 0.05-g powder for each sample was transferred into 0.5-ml extraction solution containing methanol/water/hydrochloric acid (799:200:1, v/v/v) and sequentially vortexed for 10 min, subjected to ultrasound for 10 min, and centrifuged for 3 min at 12,000 g under 4°C. After repeating the extractions twice, the two supernatants were collected and filtrated (PTFE, 0.22 μm; Anpel) before LC–MS/MS analysis.

The sample extracts of cherry were performed using an LC-ESI-MS/MS system (UPLC, Shim-pack UFLC SHIMADZU CBM30A system^1^; MS, Applied Biosystems 6,500 Triple Quadrupole, www.appliedbiosystems.com.cn/) equipped with a Waters ACQUITY BEH C18 column (1.7 μm, 2.1 mm × 100 mm). The analytical conditions were as follows: Column temperature, 40°C; flow rate, 0.35 ml/min; injection volume, 2.0 μl. The solvent system was water (0.1% formic acid; phase A) and methanol (0.1% formic acid, phase B). The gradient program for phase A/phase B was 95:5 (v/v) at 0 min, 50:50 (v/v) at 6 min, 5:95 (v/v) at 12 min, hold for 2 min, 95:5 (v/v) at 14 min, hold for 2 min. The effluent was alternatively connected to an ESI-triple quadrupole-linear ion trap (Q TRAP)-MS system. Linear ion trap (LIT) and triple quadrupole (QQQ) scans were acquired using a triple quadrupole-linear ion trap mass spectrometer (Q TRAP), API 6500 Q TRAP UPLC/MS/MS System, equipped with an ESI Turbo Ion-Spray interface, operating in positive and negative ion mode, and controlled using the Analyst 1.6.3 software (AB Sciex). The electrospray ionization (ESI) source operation parameters were as follows: Ion source, turbo spray; source temperature, 550°C; ion spray voltage (IS), 5.5 kV (positive ion mode); curtain gas, 35 psi; the collision gas (CAD), high. In addition, with further optimization of the de-clustering potential (DP) and collision energy (CE), the DP and CE of single multiple-reaction monitoring (MRM) conversion were obtained. A specific set of MRM transitions were monitored, according to the metabolites eluted within each period. The MRM for each line was performed in triplicate, and three spears were used for each repeat. Metabolite profiling and metabolomics data analyses were conducted by Metware Biotechnology Co., Ltd. (Wuhan, China).

### RNA-Seq Analysis

RNA-seq and bioinformatics analyses were conducted by Personal Biotechnology Co. Ltd. (Shanghai, China). Total RNA was extracted from frozen red and white cherry fruits using an RNAprep Pure Plant Plus Kit (TIANGEN, Beijing, China), according to the manufacturer’s instructions. Three biological repeats were prepared for each sample. The sequencing library construction of samples was generated using a TruSeq RNA Sample Preparation Kit (Illumina, San Diego, CA, United States), and the library preparations were sequenced on the Illumina HiSeq X platform. The reference genome database and gene annotation files were extracted from Genome Database for Rosaceae.^2^ The reference genome index was constructed using Bowtie2 (2.2.6), and the filtered reads were aligned to the reference genome using Tophat (2.0.14). HTSeq (0.9.1) was applied to calculate the counts of mapped reads at gene levels, and the gene expression levels are expressed in terms of FPKM (fragments per kilobase per million fragments). The DEGs were analyzed using DESeq (1.30.0) with the screening conditions |log_2_Fold Change| > 1 and value of *p* < 0.05. Subsequently, the R Pheatmap (1.0.8) software package was applied to conduct clustering analysis of all differential genes. RNA-seq analysis was performed with three biological replicates for each point.

### Correlation Analysis of Transcriptomic and Metabolomic Data

The differentially expressed metabolites (DEMs) were screened with expression difference multiple |log_2_Fold Change| > 2 and significant value of *p* <0.05. To observe the changes and associations of DEMs and significant DEGs, 11 DEMs and significant DEGs (|log_2_Fold Change| > 2, *p* < 0.05 and FPKM > 2) in the WP vs. RP comparison were used to describe a correlation network diagram with Pearson correlation coefficient (PCC) > 0.90. PCC analysis of the genes and metabolites was conducted using the cor function in the R package. The correlation network plot of DEMs and DEGs was visualized using Cytoscape (v3.7.2, United States). The volcano plot was constructed using OmicStudio tools at https://www.omicstudio.cn/tool. Gene Ontology (GO) enrichment analysis was performed using an online platform.^3^

### Real-Time PCR Analysis

Total RNA was also extracted using the RNAprep Pure Plant Plus Kit (TIANGEN, Beijing, China), and cDNA synthesis was carried out using HiScript III-RT SuperMix for the qPCR kit (Vazyme, Nanjing, China), according to our previous study (Yang et al., 2021a). In order to study the transcriptional patterns of various genes, real-time PCR was analyzed using the Bio-Rad CFX Connect Real-Time System. The primers for genes were designed using primer3,^4^ which are given in Supplementary Table 1. The melting curve and PCR product re-sequencing were used to double-check the specificity of various primers. We used the *actin* (GenBank no. FJ560908) as the housekeeping gene. All reactions were conducted using a ChamQ Universal SYBR qPCR Master Mix kit (Vazyme, Nanjing, China) in a total sample volume of 20 μl (10 μl of SYBR Premix; 0.5 μl of forward primer, 10 μM; 0.5 μl of reverse primer, 10 μM; 1.0 μl of cDNA template, 20 ng; 8.0 μl of ddH_2_O) were initiated with a preliminary step of 30 s at 95°C, followed by 40 cycles of 95°C for 5 s and 60°C for 30 s. Relative gene transcription levels were calculated using the 2^−∆Ct^ method. Three independent biological replicates were analyzed for each sampling point.

### Statistical Analysis

The statistical significance was determined with Tukey’s test, and the significance is expressed by different letters above the error bar. The heatmap was drawn by the TBtools software. Figures were drawn using the Origin 8.0 software (OriginLab Corp., Northampton, MA, United States).

## Results

### Physiological Indices in Red and White Fruits of *Prunus tomentosa*

**Figure 1:**
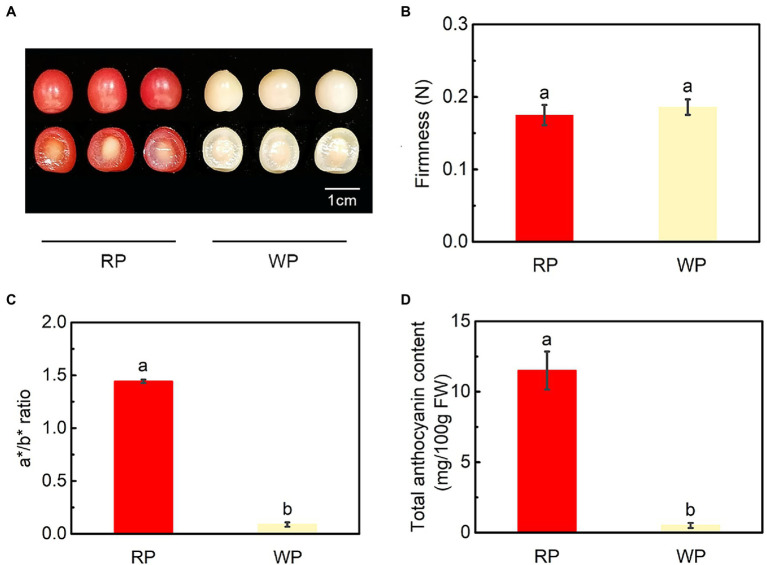
Phenotypes and physiological changes of red and white fruit of *Prunus tomentosa* in mature stage. **(A)** Phenotypes of red and white *P. tomentosa*; **(B)** Fruit firmness (N); **(C)** Changes in a^*^/b^*^ ratio; **(D)** Total anthocyanin content of cherry fruit. RP, red-color cultivar of *P. tomentosa*; WP, white-color cultivar of *P. tomentosa*. Error bars in **(B–D)** represent ± SE from three replicates. Tukey’s test significant at *p* < 0.05 is indicated by a and b.

The color of red and white *P. tomentosa* fruits showed an obvious difference, with orange-red pigmentation in RP and creamy white in WP fruit at mature stage (Figure 1A). The firmness of RP and WP fruits were 0.18 N and 0.19 N, respectively, indicating that fruit firmness was similar in red and white *P. tomentosa* (Figure 1B). In addition, the a^*^/b^*^ ratio was considered as an indicator of fruit color; here, it was obviously higher in RP than in WP (Figure 1C). Consistent with the color variance of the fruit, total anthocyanin content was 22-fold higher in RP (11.50 mg/100 g FW) than in WP (0.51 mg/100 g FW) fruits at the mature stage (Figure 1D). Accordingly, the anthocyanin accumulation was enhanced in the red cherries.

### Identification and Comparison of Anthocyanin and Procyanidin Compounds

**Figure 2:**
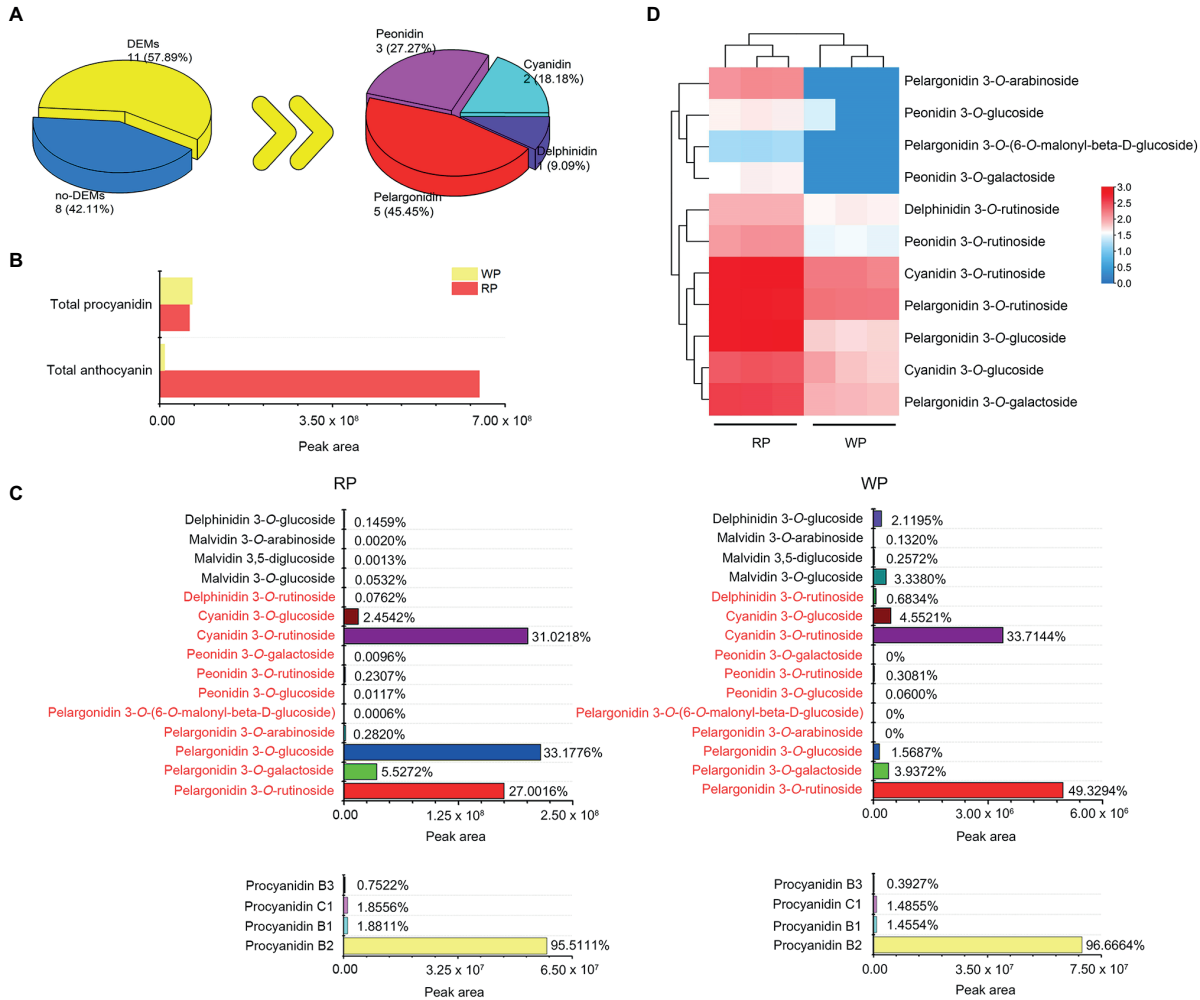
Metabolomics profiles for cherry fruits of *Prunus tomentosa*. **(A)** Statistics of the expressed metabolites and differentially expressed metabolites (DEMs: |log_2_Fold Change| > 2 and value of *p* <0.05); **(B)** The relative contents of total procyanidin and total anthocyanin from metabolome data. The relative content of metabolites is expressed by peak area; **(C)** The relative contents and proportion of anthocyanin (upper) and procyanidin (lower) compounds in red and white fruits of *P. tomentosa*. The red font indicates the 11 DEMs; **(D)** Heatmap of the 11 DEMs at mature stage. Color indicates the relative content of each DEM, from blue (low) to red (high).

To gain further insight into the coloration mechanism of *P. tomentosa*, LC–MS/MS analysis of anthocyanin compounds was applied to mature fruit. In this study, a total of 15 anthocyanins and four procyanidins were isolated from the RP and WP samples (Supplementary Table 2). Among them, 11 differentially expressed metabolites were identified based on the thresholds |log_2_Fold Change| > 2 and value of *p* < 0.05, including five pelargonidins (pelargonidin 3-*O*-arabinoside, pelargonidin 3-*O*-glucoside, pelargonidin 3-*O*-(6-*O*-malonyl-beta-D-glucoside, pelargonidin 3-*O*-galactoside, and pelargonidin 3-*O*-rutinoside), three peonidins (peonidin 3-*O*-galactoside, peonidin 3-*O*-rutinoside, and peonidin 3-*O*-glucoside), two cyanidins (cyanidin 3-*O*-rutinoside and cyanidin 3-*O*-glucoside), and one delphinidin (delphinidin 3-*O*-rutinoside); see Figure 2A, Supplementary Table 2. The amounts of some pelargonidin-, peonidin-, and cyanidin-based anthocyanins were considered sources of the orange–red color. The RP fruits accumulated much more anthocyanins than WP fruits, showing 64-fold greater accumulation (Figure 2B). However, there was no significant difference in total procyanidins (Figure 2B), among which procyanidin B2 (96.6664% in RP, 95.5111% in WP) was the major procyanidin in both RP and WP fruits (Figure 2C). In red *P. tomentosa*, pelargonidin 3-*O*-glucoside (33.1776%), cyanidin 3-*O*-rutinoside (31.0218%), and pelargonidin 3-*O*-rutinoside (27.0016%) were the highest accumulated anthocyanins, accounting for 91.201% of the total anthocyanins (Figure 2C). In white *P. tomentosa*, the proportion of pelargonidin 3-*O*-rutinoside was the largest, which accounted for approximately half (49.3294%), followed by cyanidin 3-*O*-rutinoside (33.7144%; Figure 2C). In the RP and WP comparison, the greatest different metabolite was pelargonidin 3-*O*-glucoside, being 1,362-fold higher in the RP samples than in the WP samples (Figure 2C; Supplementary Table 2). A heatmap based on relative expression levels of the 11 DEMs is shown in Figure 2D, all of which were more abundant in RP compared with WP fruits. Therefore, it appears that the fruit color differences between RP and WP were not only dependent on their total anthocyanin contents, but also on their anthocyanin compositions.

### Transcriptomic Analysis of RP and WP Fruits

**Figure 3:**
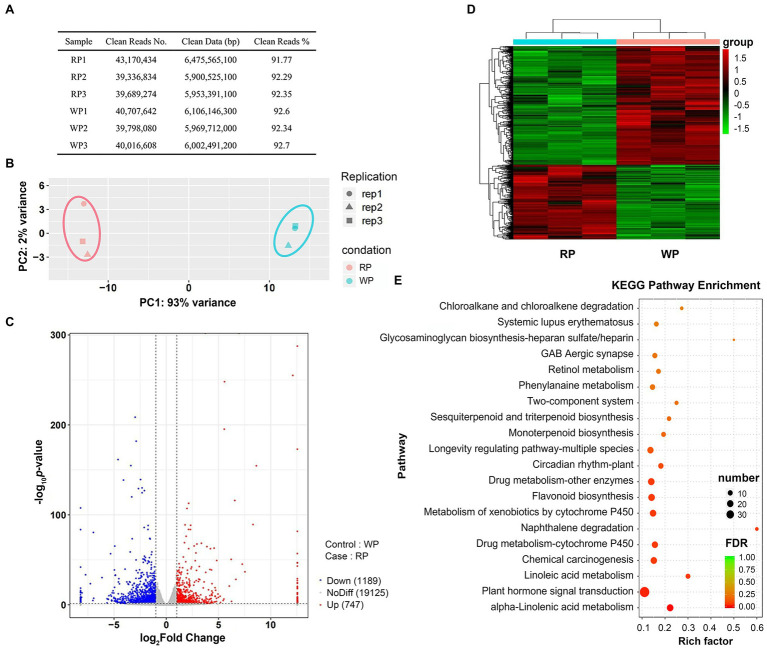
The RNA-seq expression profiles of *Prunus tomentosa* in mature stage. **(A)** Statistics of sequencing data filtering in RP and WP samples; **(B)** Principal component analysis (PCA) of genes identified in *P. tomentosa* fruit at mature stage; **(C)** Volcano plots of the gene transcription profile in WP and RP libraries. Red dots represent the upregulated genes, blue dots represent the downregulated genes; **(D)** Heatmap of all differential expression genes (DEG: |log_2_Fold Change| > 1 and value of *p* <0.05). Color indicates the expression level of each gene, from green (low) to red (high); **(E)** KEGG enrichment analysis with the top 20 KEGG pathways in the DEGs in RP vs. WP comparison.

To further investigate the molecular mechanisms of anthocyanin accumulation in *P. tomentosa* fruits, RNA-seq analysis was carried out to sequence the transcripts. After sequencing the cDNA libraries, removing low-quality sequences and adapters, the number of clean reads ranged from 39,336,834 to 43,170,434 for six libraries for RP and WP, and the clean data ranged from 5,900,525,100 to 6,475,565,100 bp. Clean reads in all samples were at least 91.77% of the total (Figure 3A). Principal component analysis (PCA) was implemented to show the distinctly separate groups among the two samples. Here, the first and second principal components (PC1 and PC2) for the cherries explained 93% and 2% of the variance, respectively (Figure 3B). Thus, PC1 was the dominant component in this study, and the RP and WP samples were clearly separated according to PC1 (Figure 3B). An FPKM value threshold of |log_2_Fold Change| > 1 and value of *p* < 0.05 were used for screening DEGs. A total of 1,936 DEGs were identified, including 747 upregulated genes and 1,189 dowregulated genes, in the WP vs. RP comparison (Figure 3C). An overview of all of the 1,936 DEGs is provided, in terms of a volcano plot and heatmap, in Figures 3C,D, respectively. Among these DEGs, the Kyoto Encyclopedia of Genes and Genomes (KEGG) pathway enrichment analysis revealed that they were significantly enriched in a number of metabolic pathways, including plant hormone signal transduction (ko04075), flavonoid biosynthesis (ko00941), and alpha-Linolenic acid metabolism (ko00592) pathways (Figure 3E).

### Combined Transcriptome and Metabolome Analysis of DEMs and DEGs

**Figure 4:**
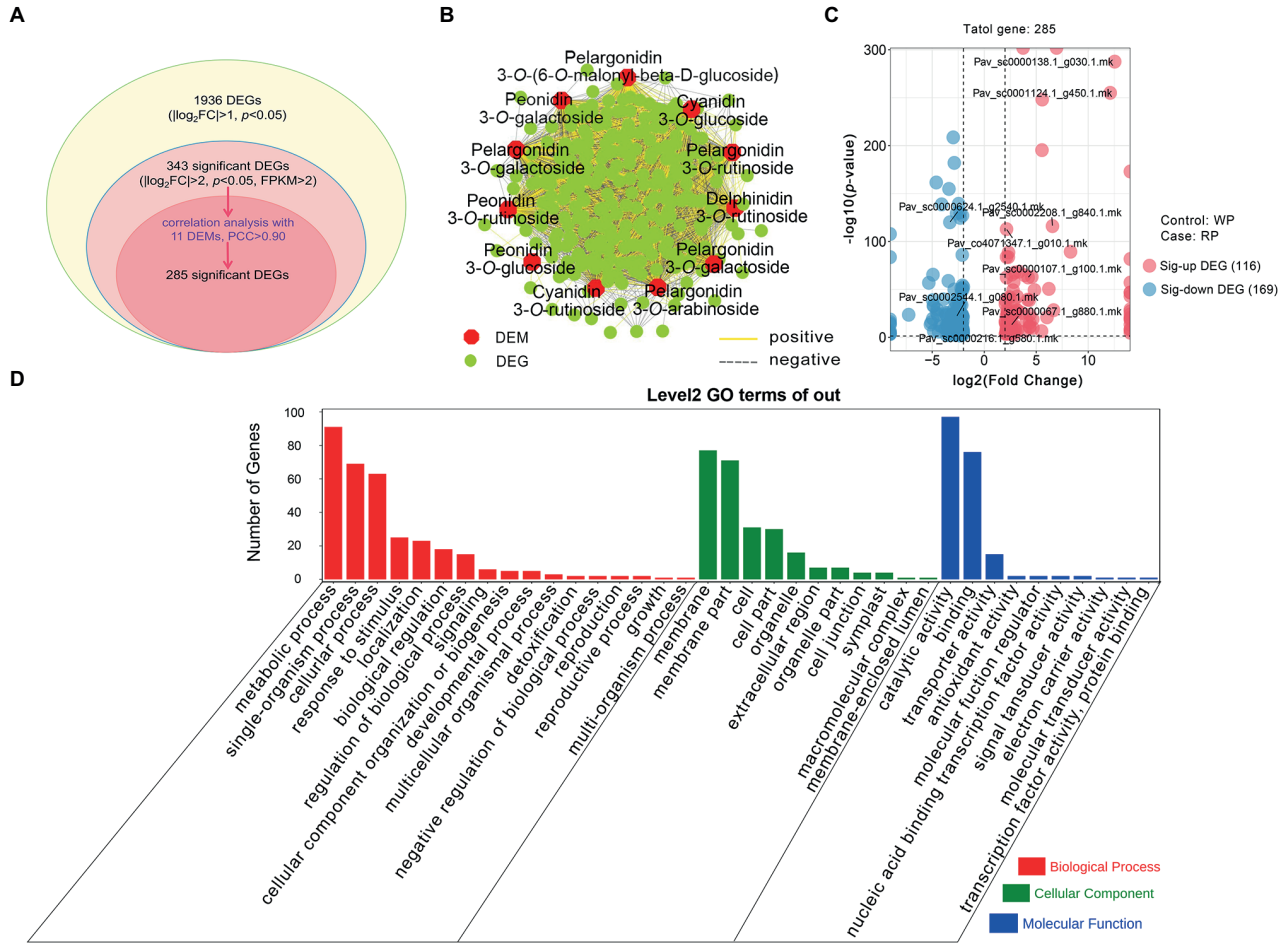
Comparative analysis of the transcriptomic and metabolomic data from RP and WP fruits. **(A)** Statistics of the DEGs and significant DEGs. FC: Fold Change, *p,* value of *p*; **(B)** Connection network for association analysis of DEMs (|log_2_Fold Change| > 2 and value of *p* < 0.05) and significant DEGs (|log_2_Fold Change| > 2, value of *p* < 0.05, and FPKM > 2) with Pearson correlation coefficient (PCC) > 0.90. The red regular hexagon and green circle represent DEMs and DEGs, respectively. The yellow and gray edges represent positive and negative correlations, respectively; **(C)** Volcano plots of 285 significant DEGs. Red dots represent the upregulated genes, blue dots represent the downregulated genes. Sig-up DEG: significant upregulated DEG, Sig-down DEG: significant downregulated DEG; **(D)** Gene ontology (GO) functional categories assigned to 285 significant DEGs.

Based on the RNA-seq results, we improved the thresholds for screening significant DEGs with |log_2_Fold Change| > 2, value of *p* < 0.05, and FPKM > 2. In total, 343 significant DEGs emerged from all 1,936 DEGs. Then, according to the threshold of Pearson’s correlation coefficient (PCC) > 0.90 between 11 DEMs (from the metabolome) and 343 significant DEGs, a total of 285 significant DEGs were obtained (Figure 4A). The correlation analysis showed that each anthocyanin was closely related to multiple genes, indicating that the anthocyanin accumulation process is regulated by multiple genes, rather than just a single gene (Figure 4B). Among the 285 genes, 116 genes were significantly upregulated and 169 genes were downregulated in RP compared with WP (Figure 4C). In order to provide insight into the functions of these significant DEGs, GO enrichment analysis based on term classification was implemented. The 285 co-regulated DEGs were annotated and classified into three major categories, including biological process (BP), cellular component (CC), and molecular function (MF); see Figure 4D. Among these DEGs, metabolic process (GO:0008152, 91 DEGs), membrane (GO:0016020, 77 DEGs), and catalytic activity (GO:0003824, 97 DEGs) were the three most-enriched terms (Figure 4D). These results further indicate that these significant DEGs are likely to participate in the formation of anthocyanin metabolites, either directly or indirectly.

### Candidate Significant DEGs Involved in Anthocyanin Synthesis, Transport, and Degradation

**Figure 5:**
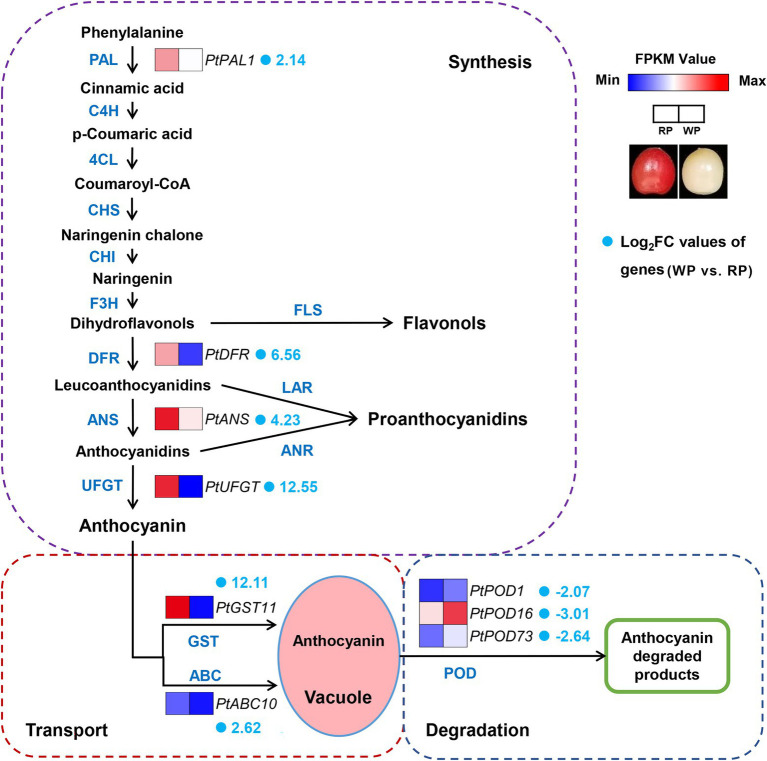
Expression pattern of the candidate key significant DEGs involved in anthocyanin synthesis, transport, and degradation pathways in *Prunus tomentosa*. The color scale from blue to red represents the FPKM values from low to high. The fold number behind gene names represents the log_2_Fold Change values of genes (WP vs. RP).

In plants, anthocyanin biosynthetic pathways have been widely studied, where anthocyanin transport and degradation also influence the final anthocyanin content. Thus, we focus on anthocyanin biosynthesis, transport, and degradation pathways in this study. The abundances of significant DEGs related to anthocyanin biosynthesis, transport, and degradation were further compared between the RP and WP transcriptomes, in order to identify candidate key enzyme-encoding genes involved in the coloration of the fruit. The results demonstrated a total of nine significant DEGs, which exhibited obvious different transcript abundances in the RP samples than in WP, consisting of four anthocyanin biosynthesis genes (*Pav_co4071347.1_g010.1.mk*, named *PtPAL1; Pav_sc0002208.1_g840.1.mk*, named *PtDFR; Pav_sc0000107.1_g100.1.mk*, named *PtANS; Pav_sc0000138.1_g030.1.mk*, named *PtUFGT*), two transport genes (*Pav_sc0001124.1_g450.1.mk*, named *PtGST11; Pav_sc0000067.1_g880.1.mk*, named *PtABC10*), and three degradation-related genes (*Pav_sc0000216.1_g580.1.mk*, named *PtPOD1*; *Pav_sc0000624.1_g2540.1.mk*, named *PtPOD16*; *Pav_sc0002544.1_g080.1.mk*, *PtPOD73*); see Figure 5; Supplementary Table 3. The transcript levels of these genes, based on the FPKM values from RNA-Seq data, are involved in anthocyanin synthesis, transport, and degradation pathways (Figure 5). Interestingly, the abundances of four structural genes in the anthocyanin biosynthesis pathway and *PtGST11/PtABC10* (which are involved in the transport process) were significantly higher in RP compared with WP fruit (Figure 5), consistent with the high content of anthocyanin metabolites in red *P. tomentosa* fruit. To the contrary, three *PODs* (*PtPOD1/16/73*) encoding enzymes involved in anthocyanin degradation were expressed at a lower level in RP than WP fruit (Figure 5). In the anthocyanin synthesis part, except for one upstream structural gene (*PtPAL1*) the others were all involved in the late stage of the anthocyanin synthesis pathway cascade, which function specifically in anthocyanin biosynthesis. Among them, *PtUFGT* showed the most dramatic change in expression level, with 12.55-fold higher log_2_Fold Change values in RP than in WP fruit (Figure 5). Moreover, GST genes and ABC transporter genes may be involved in the transport of anthocyanins to vacuoles; in this study, *PtGST11* showed 12.11-fold higher log_2_Fold Change values in RP, being the second-most significant DEG in the anthocyanin accumulation process (Figure 5).

**Figure 6:**
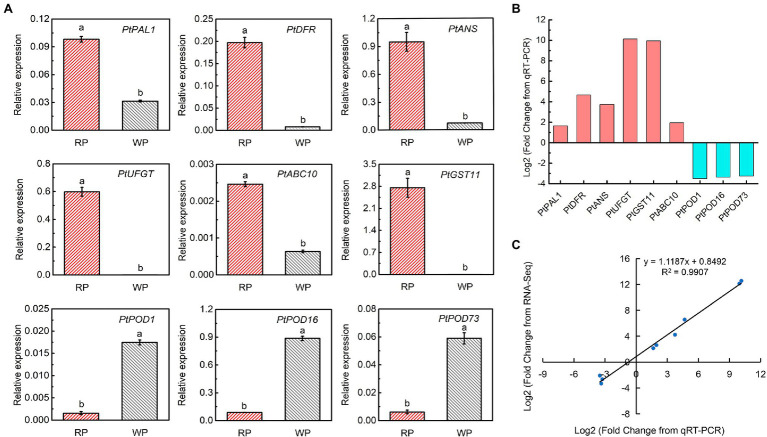
Expression analysis of the key significant DEGs related to the anthocyanin accumulation. **(A)** Gene expression of candidate key significant DEGs involved in anthocyanin synthesis, transport, and degradation. Tukey’s test significant at *p* < 0.05 indicated by a and b. **(B)** Log_2_Fold Change of differentially expressed anthocyanin-related genes between WP and RP from real-time PCR. **(C)** Correlation analysis based on RNA-seq data and real-time PCR.

To further verify the reliability of the transcriptomic data and examine the expression of the candidate DEGs, real-time PCR was performed to measure the transcript abundances of nine significant DEGs between RP and WP fruits (Figure 6A), among them, *PtANS* and *PtPOD16* showed high transcript abundance in red and white *P. tomentosa* fruit (Figure 6A). Furthermore, the most significantly different genes were *PtUFGT* and *PtGST11*, with approximately 10-fold log_2_Fold Change values in the WP vs. RP comparison (Figure 6B). These crucial genes in anthocyanin synthesis and transport pathways had similar expression pattern, and appear to play vital roles in anthocyanin formation and accumulation in *P. tomentosa*, whereas PODs took on exactly opposite trend, which may be responsible for catalyzing the anthocyanins (Figures 6A,B). Finally, a correlation analysis exhibited a significant correlation coefficient of 0.9907 between RNA-Seq and real-time PCR (Figure 6C), suggesting that the transcriptome data accurately consistent with the expression of anthocyanin-related genes in *P. tomentosa* fruit.

## Discussion

### Color Variation and Anthocyanin Content Difference Between RP and WP Fruits

Color mutation is a quite important phenotype of horticultural products, playing a key role in plant breeding and horticulture. Moreover, color is one of the most efficient indicators for evaluating fruit quality and maturity. In the mature stage, the color of cherry fruits mainly present as dark red, such as in “Hong Deng” (*P. avium*), “Black Pearl” (*P. avium*), “Santina” (*P. avium*), “Kitayanka” (*P. avium*), “Irema BS” (*P. avium*), “Werdersche braune” (*P. avium*), “Erdi bottermo” (*P. cerasus*), and “Aode” (*P. cerasus*; Liu et al., 2013; Cao et al., 2015; Starkevič et al., 2015; Zhang et al., 2021; Yang et al., 2021b); bright red, such as in “Lapins” (*P. avium*), “Duan bing” (*P. cerasus*), and “Black peel” (*P. cerasus*; Cao et al., 2015; Starkevič et al., 2015); yellow-and-bright-red bicolor, such as in “Caihong” (*P. avium*) and “Rainier” (*P. avium*; Liu et al., 2013; Jin et al., 2016); or yellow, such as in “Big Dragon” (*P. avium*) and “Belobokaya rannyaya” (*P. avium*; Starkevič et al., 2015; Jin et al., 2016). Interestingly, mature *P. tomentosa* fruits present as orange–red or creamy white (Figure 1A), greatly different from other cherries (Cao et al., 2015). The formation of red-flesh and red-peel in fruit is dependent on the anthocyanin content and composition, as well as the associated accumulation patterns. Usually, red pigment accumulation and anthocyanin content increase reach their deepest and the highest at the mature stage (Liu et al., 2013; Shen et al., 2014; Starkevič et al., 2015; Jin et al., 2016; Yang et al., 2021a). These suggest that anthocyanin may also play an important role in fruit ripening (Carbone et al., 2019; Yang et al., 2021b). In this study, the a^*/^b^*^ value ratio was significantly higher in RP than WP fruits at mature stage, which is consistent with the color difference in the appearance of red and white *P. tomentosa* cherries (Figures 1A,C). Moreover, similar significant differences also appeared in the total anthocyanins content (Figures 1D, 2B). These obvious differences between WP and RP fruit indicate that the color formation in red *P. tomentosa* cherries is induced by increased anthocyanin levels, and the total anthocyanin content is responsible for the difference in fruit color.

### Comparison of Anthocyanin Compounds in RP and WP Fruits

A wide variety of anthocyanin compounds have been found in plants, and previous studies have indicated that cyanidin, pelargonidin, delphinidin, peonidin, petunidin, and malvidin are the most common anthocyanins (He and Giusti, 2010; Jaakola, 2013). Among them, pelargonidin has been reported as representing an orange–red/orange–salmon color, while cyanidin and peonidin represent a bluish–red/magenta/crimson color (Bueno et al., 2012; Ali et al., 2016). In this study, a total of 15 anthocyanin metabolites and four procyanidins were identified in white and red *P. tomentosa* fruit, based on LC–MS/MS (Supplementary Table 2), which contained 11 DEMs, including five pelargonidins, three peonidins, two cyanidins, and one delphinidin (Figures 2A–D). To date, the anthocyanin profile and components have been determined in many cherries. Cyanidin and its glycoside derivatives have been reported as the primary anthocyanins in red-colored cherries (Homoki et al., 2016; Acero et al., 2019; Blando and Oomah, 2019; Brozdowski et al., 2021). In the extract of sweet cherry peels, five anthocyanins have been detected, and cyanidin 3-rutinoside was found to be the most prominent (Turturică et al., 2016). Meanwhile, cyanidin 3-*O*-glucoside and cyanidin 3-*O*-rutinoside are the major anthocyanins in sweet cherry fruits (Acero et al., 2019). Cyanidin 3-*O*-rutinoside has been shown to represent a preliminary index for grouping similar cherry cultivars and colors (Jia et al., 2019). In black cherries, cyanidin 3-rutinoside and cyanidin 3-glucoside have also been reported as two dominant anthocyanidin compounds (Brozdowski et al., 2021). In tart cherries, the most enriched three anthocyanidins were cyanidin 3-glucosylrutinoside, cyanidin 3-rutinoside, and cyanidin 3-glucoside (Homoki et al., 2016). In *P. tomentosa*, “Red” had a different anthocyanin profile compared to other species, with three totally different compounds identified (pelargonidin 3-rutinoside, cyanidin 3-rutinoside, and pelargonidin 3-glucoside), where pelarogonidin-3-rutinoside was the dominant compound; in contrast, these anthocyanins were not detected in “White” fruits (Cao et al., 2015). However, in the present study, a total of 12 anthocyanidins were detected in white *P. tomentosa*, although their contents were much lower, compared to that in red *P. tomentosa* (Figures 2B,C); therefore, this is the first time that anthocyanins have been identified in white *P. tomentosa*. Pelargonidin 3-*O*-glucoside (33.1776%), cyanidin 3-*O*-rutinoside (31.0218%), and pelargonidin 3-*O*-rutinoside (27.0016%) were the three dominant anthocyanin compounds in red *P. tomentosa* (Figure 2C). Thus, the anthocyanin compounds in red *P. tomentosa* were generally similar to those previously reported by Cao et al. (2015); however, our results were more extensive, abundant, and detailed, considering the anthocyanidin profile in both RP and WP, which may be derived from relevant technological advances. Meanwhile, although the anthocyanin content level was limited in the white cherries of *P. tomentosa*, they contained all of the core anthocyanidin components detected in red *P. tomentosa*, including the glycoside derivatives of pelargonidin, cyanidin, and peonidin, of which pelargonidin 3-*O*-glucoside was extremely significant different between WP and RP (1361.97-fold, Figures 2C,D; Supplementary Table 2). These results suggest that the color appearance of “Red” *P. tomentosa* (orange–red) and white *P. tomentosa* (creamy white) is mainly determined by the total anthocyanin content, as well as the anthocyanin composition and percentage (e.g., pelargonidin 3-*O*-glucoside).

### Combined Metabolome and Transcriptome Analyses Uncover Key Candidate Genes in Anthocyanin Biosynthesis Pathway

Anthocyanin biosynthesis is co-regulated by a series of structural genes, including *PAL*, *C4H*, *4CL*, *CHS*, *CHI*, *F3H*, *DFR*, *ANS*/*LDOX*, and *UFGT*, which have been widely reported in many fruits. In sweet cherry, six anthocyanin biosynthetic genes, *PacCHS*, *PacCHI*, *PacF3H*, *PacDFR*, *PacANS*, and *PacUFGT* have been identified and believed to participate in anthocyanin biosynthesis (Liu et al., 2013; Shen et al., 2014). Our previous study has revealed an ANS, *PacANS*, as a pivotal anthocyanin biosynthetic gene in “Hong Deng” through RNA-seq and WGCNA (Yang et al., 2021a), which is in the same gene family as *PtANS* in this study. Recent technical advancements in metabolome and transcriptome analyses have provided effective and reliable ways to identify new metabolites and genes, as well as to elucidate complex secondary metabolic processes in plants. In this study, we carried out metabolome and transcriptome analyses using red and white *P. tomentosa* fruits, and accessed enormous quantities of data. After integrative analyses, four key anthocyanin biosynthetic genes (*PtPAL1*, *PtDFR*, *PtANS*, and *PtUFGT*) were identified, indicating the reliability of the analysis methods. The transcriptome results, together with qRT-PCR, revealed that the expression levels of *PtPAL1*, *PtDFR*, *PtANS*, and *PtUFGT* were significantly higher in red *P. tomentosa* than in white *P. tomentosa*; among them, *PtANS* showed the highest expression level, while *PtUFGT* exhibited the biggest difference (Figures 5, 6). These results were consistent with the detected differences in WP and RP anthocyanin levels (Figures 1D, 2B), suggesting that *PtPAL1*, *PtDFR*, *PtANS*, and *PtUFGT* are potential key genes controlling anthocyanin biosynthesis for the red color appearance in *P. tomentosa*, especially *PtANS* and *PtUFGT*. Furthermore, there were other DEGs involved in anthocyanin biosynthesis bioprocesses, such as *Pav_sc0000636.1_g260.1.mk* (*Pt4CL*), *Pav_sc0000045.1_g280.1.mk* (*PtCHS*), and *Pav_sc0000030.1_g1340.1.mk* (*PtFLS*; data not shown), which were eliminated due to our strict and comprehensive screening conditions. However, we cannot deny the potential possibility that these may also participate in or affect the synthesis of anthocyanins.

### Anthocyanin Transport and Degradation Structural Genes Influence Anthocyanins Formation

Compared to reports available on anthocyanin biosynthesis, research on anthocyanin transport or degradation is scarce, particularly for cherries. In this study, we identified two transport (*PtGST11*, *PtABC10*) and three degradation (*PtPOD1/16/73*) genes in *P. tomentosa* (Figure 5). GSTs are a large gene family in plants, and ubiquitously participate in flavonoid metabolism, biotic and abiotic stress responses (Loyall et al., 2000; Moons, 2005). Among various functions, they are also known to mediate the transport and accumulation of anthocyanins, and the absence of GSTs often results in an anthocyanin-less phenotype with reduced pigmentation (Marrs et al., 1995; Kitamura et al., 2004; Luo et al., 2018; Jiang et al., 2019; Zhao et al., 2020). In addition, ABC transporters are also involved in the transport and accumulation of anthocyanins (Goodman et al., 2004). According to our results, the transcriptional expression levels of *PtGST11* and *PtABC10* in RP were significantly higher than in WP (Figures 5, 6), especially *PtGST11*, corresponding to the change in anthocyanin levels. Therefore, the upregulation of *PtGST11* and *PtABC10* may play positive roles in anthocyanin transport and result in the accumulation of anthocyanins in vacuoles. The effect of *PtGST11* was obviously stronger, implying its indispensable effect in anthocyanin transport and accumulation.

Some recent studies have confirmed that PODs are candidates for anthocyanin degradation in plants, such as *BcPrx01* in *Brunfelsia calycina* flowers (Zipor et al., 2015), *POD3*/*6*/*63* in strawberry (Zhang et al., 2019), and *MpPOD1*/*8*/*9* in apple (Rehman et al., 2017). In the present study, three *POD* genes (*PtPOD1*, *PtPOD16,* and *PtPOD73*) were identified among the 285 significant DEGs, the transcriptional expression levels of which were all higher in white *P. tomentosa* fruits (Figures 5, 6). The results suggested that anthocyanin degradation may be an important factor for the formation of the creamy white color of white *P. tomentosa* fruit, and *PtPOD1/16/73* may have a considerable contribution. Evidently, the anthocyanin levels differed substantially between WP and RP, which may be determined by the direct expression of the structural genes involved in anthocyanin biosynthesis, transport, and degradation.

## Conclusion

In conclusion, we used orange–red and creamy white cherries of *P. tomentosa* as materials to confirm the different mechanisms underlying their different color patterns and found that the anthocyanin content was significantly higher in red *P. tomentosa* compared with the creamy white fruits. Then, a total of 15 anthocyanins and four procyanidins were identified by LC–MS/MS, including 11 significant different anthocyanins in the RP vs. WP comparison. Pelargonidin 3-*O*-glucoside, cyanidin 3-*O*-rutinoside, and pelargonidin 3-*O*-rutinoside were the major anthocyanins in red *P. tomentosa* fruit, while pelargonidin 3-*O*-rutinoside and cyanidin 3-*O*-rutinoside were the major anthocyanins in white *P. tomentosa* fruit. Based on metabolome and transcriptome analyses, nine candidate genes closely related to anthocyanin accumulation were selected from 285 significant DEGs, including four biosynthetic genes (*PtPAL1*, *PtDFR*, *PtANS, PtUFGT*), two transporter genes (*PtGST11*, *PtABC10*), and three degradation genes (*PtPOD1*, *PtPOD16, PtPOD73*). Moreover, the biosynthetic and transporter genes were significantly more highly expressed in red *P. tomentosa* fruits, compared to white *P. tomentosa* fruits, while the expression trend of the degradation genes was opposite. The present study not only provides novel insights for understanding the DEMs which may determine the formation of anthocyanins, but we also identified nine candidate key genes involved in anthocyanin biosynthesis, transport, and degradation pathways. Future work is required to investigate the underlying molecular mechanisms and candidate regulators of anthocyanin accumulation in cherries.

## Data Availability Statement

The datasets presented in this study can be found in online repositories. The names of the repository/repositories and accession number(s) can be found in the article/Supplementary Material.

## Author Contributions

JL, HG, and AZ designed the study. HY, SJ, CT, and NC performed the experiments. AZ, HY, and SJ analyzed the data. JL, HG, AZ, and HY wrote the manuscript. All authors contributed to the article and approved the submitted version.

## Funding

This research was supported by the National Natural Science Foundation of China (31901737), the Special Fund for Innovation Teams of Fruit Trees in Agricultural Technology System of Shandong Province, China (SDAIT-06-02), and the Natural Science Foundation of Shandong Province (ZR2021QC157). The funders had no role in the material creation, designing the study, analysis data and in writing the manuscript.

## Conflict of Interest

The authors declare that the research was conducted in the absence of any commercial or financial relationships that could be construed as a potential conflict of interest.

## Publisher’s Note

All claims expressed in this article are solely those of the authors and do not necessarily represent those of their affiliated organizations, or those of the publisher, the editors and the reviewers. Any product that may be evaluated in this article, or claim that may be made by its manufacturer, is not guaranteed or endorsed by the publisher.

## Footnotes

^1^ www.shimadzu.com.cn/

^2^ https://www.rosaceae.org/species/prunus/all

^3^ https://www.omicshare.com/tools

^4^ https://bioinfo.ut.ee/primer3-0.4.0/
