# Supplementary material for: Metabolome and Transcriptome Analyses of Anthocyanin Accumulation Mechanisms Reveal Metabolite Variations and Key Candidate Genes Involved in the Pigmentation of Prunus tomentosa Thunb. Cherry Fruit
Source: Front Plant Sci. 2022 Jun 29;13:938908. doi: 10.3389/fpls.2022.938908 (PMC9277446; doi:10.3389/fpls.2022.938908)
Supplement: Supplementary file 1 [file Data_Sheet_1.docx]

Supplementary Material

**Supplementary Table 1.** Primers for real-time PCR.

| **Gene** | **Forward (5’ to 3’)** | **Reverse (5’ to 3’)** |
| --- | --- | --- |
| ***PtPAL1*** | GTTGGGTGCAGAGTACCTAACAG | GAAATGCTAACAGATCGGAAGTG |
| ***PtDFR*** | CCGAGTACAATATTCCCACAAAG | TCCTCCAAGCTGTATTTGAACTC |
| ***PtANS*** | GCCAAGTGTGTTCCAAATTCCA | GATCTTCTCCTTTGGTGGCTCA |
| ***PtUFGT*** | ACTGTTGAGGATGGGGTTTTTAC | GTGCGAGTAGTTTAACGGTGTTT |
| ***PtGST11*** | GGAGAAGGTGTTCGATGTTTATG | AGCATTCACATTCTTCCTCTCAG |
| ***PtABC10*** | GGCTTATGGGTTCCTTCTTTCT | TCAGGAAACACTCAAGTGGGTA |
| ***PtPOD1*** | CTTCCTTTCTCAACTGCAATCAC | CATAACTTCTGATCCGACTCCAG |
| ***PtPOD16*** | TGTCTACTTCCAGAACCTTCAGC | TCACCTTGATTTCCAGTCAGAAC |
| ***PtPOD73*** | CATATGCAACTCAACTCCAACAG | AGGACCTGGTCTGAGGTAAAAAG |

**Supplementary Table 2.** Anthocyanin and procyanidin compounds in red and white fruits of *Prunus tomentosa*.

| **Compound** | **Molecular Weight (Da)** | | **WP** | | **RP** | | ***p*-value** | | **Fold Change** | | **type** | |  |
| --- | --- | --- | --- | --- | --- | --- | --- | --- | --- | --- | --- | --- | --- |
| Pelargonidin 3-*O*-rutinoside | | 579.1708 | | 4.96 × 10^6^ ± 2.82 × 10^5^ | | 1.75 × 10^8^ ± 6.18 × 10^6^ | | 1.29 × 10^-3^ | | 35.2545 | | up | |
| Pelargonidin 3-*O*-galactoside | | 433.1135 | | 3.96 × 10^5^ ± 4.45 × 10^4^ | | 3.58 × 10^7^ ± 2.48 × 10^6^ | | 4.87 × 10^-3^ | | 90.4017 | | up | |
| Pelargonidin 3-*O*-glucoside | | 433.1135 | | 1.58 × 10^5^ ± 1.91 × 10^4^ | | 2.15 × 10^8^ ± 1.54 × 10^7^ | | 5.09 × 10^-3^ | | 1361.9736 | | up | |
| Pelargonidin 3-*O*-arabinoside | | 403.1029 | | 9.00 | | 1.83 × 10^6^ ± 2.25 × 10^5^ | | 1.48 × 10^-2^ | | 203000 | | up | |
| Pelargonidin 3-*O*-(6-*O*-malonyl-beta-D-glucoside) | | 519.1139 | | 9.00 | | 3.99 × 10^3^ ± 3.15 × 10^2^ | | 6.22 × 10^-3^ | | 442.8593 | | up | |
| Peonidin 3-*O*-glucoside | | 463.124 | | 6.04 × 10^3^ ± 6.03 × 10^3^ | | 7.58 × 10^4^ ± 5.56 × 10^3^ | | 1.08 × 10^-3^ | | 12.5457 | | up | |
| Peonidin 3-*O*-rutinoside | | 609.1819 | | 3.10 × 10^4^ ± 2.54 × 10^3^ | | 1.49 × 10^6^ ± 1.90 × 10^5^ | | 1.65 × 10^-2^ | | 48.2132 | | up | |
| Peonidin 3-*O*-galactoside | | 463.124 | | 9.00 | | 6.22 × 10^4^ ± 7.54 × 10^3^ | | 1.43 × 10^-2^ | | 6905.963 | | up | |
| Cyanidin 3-*O*-rutinoside | | 595.1663 | | 3.39 × 10^6^ ± 4.75 × 10^5^ | | 2.01 × 10^8^ ± 1.30 × 10^7^ | | 4.23 × 10^-3^ | | 59.2529 | | up | |
| Cyanidin 3-*O*-glucoside | | 449.1084 | | 4.58 × 10^5^ ± 2.37 × 10^5^ | | 1.59 × 10^7^ ± 1.48 × 10^6^ | | 7.78 × 10^-3^ | | 34.7184 | | up | |
| Delphinidin 3-*O*-rutinoside | | 611.1612 | | 6.88 × 10^4^ ± 5.45 × 10^3^ | | 4.94 × 10^5^ ± 1.69 × 10^4^ | | 6.25 × 10^-4^ | | 7.1845 | | up | |
| Malvidin 3-*O*-glucoside | | 493.4 | | 3.36 × 10^5^ ± 2.13 × 10^4^ | | 3.44 × 10^5^ ± 1.21 × 10^4^ | | 7.50 × 10^-1^ | | 1.0254 | | normal | |
| Malvidin 3,5-diglucoside | | 691 | | 2.59 × 10^4^ ± 1.17 × 10^3^ | | 8.31 × 10^3^ ± 2.38 × 10^2^ | | 3.27 × 10^-3^ | | 0.3212 | | normal | |
| Malvidin 3-*O*-arabinoside | | 463.4 | | 1.33 × 10^4^ ± 4.19 × 10^3^ | | 1.27 × 10^4^ ± 1.02 × 10^3^ | | 8.99 × 10^-1^ | | 0.9538 | | normal | |
| Delphinidin 3-*O*-glucoside | | 465.4 | | 2.13 × 10^5^ ± 8.02 × 10^4^ | | 9.45 × 10^5^ ± 4.62 × 10^5^ | | 2.52 × 10^-1^ | | 4.4321 | | normal | |
| **Total anthocyanin** | |  | | 1.01 × 10^7^ ± 9.81 × 10^5^ | | 6.48 × 10^8^ ± 2.70 × 10^7^ | |  | | 64.3958 | |  | |
| Procyanidin B2 | | 578.5 | | 6.40 × 10^7^ ± 6.77 × 10^6^ | | 5.58 × 10^7^ ± 3.94 × 10^6^ | | 4.80 × 10^-1^ | | 0.9025 | | normal | |
| Procyanidin B1 | | 578.5 | | 9.64 × 10^5^ ± 8.27 × 10^4^ | | 1.14 × 10^6^ ± 1.48 × 10^5^ | | 3.78 × 10^-1^ | | 1.1805 | | normal | |
| Procyanidin C1 | | 866.8 | | 9.84 × 10^5^ ± 1.88 × 10^5^ | | 1.12 × 10^6^ ± 1.21 × 10^5^ | | 5.74 × 10^-1^ | | 1.1409 | | normal | |
| Procyanidin B3 | | 578.5 | | 2.60 × 10^5^ ± 1.26 × 10^4^ | | 4.55 × 10^5^ ± 1.54 × 10^5^ | | 3.33 × 10^-1^ | | 1.7495 | | normal | |
| **Total procyanidin** | |  | | 6.62 × 10^7^ ± 7.05 × 10^6^ | | 6.05 × 10^7^ ± 4.35 × 10^6^ | |  | | 0.9134 | |  | |

Note: WP: the fruits of ‘white *P. tomentosa*’ at mature stage; RP: the fruits of ‘red *P. tomentosa*’ at mature stage. Number 9.00 represents a level so low as to barely be detectable.

**Supplementary Table 3.** The information of differentially expressed genes involved in anthocyanin synthesis, transport, and degradation.

| **Gene** | **NCBI accession** | **Genome number** | **Reference** | | **Full name** | |  |
| --- | --- | --- | --- | --- | --- | --- | --- |
| ***PtPAL1*** | XP_021804316.1 | *Pav_co4071347.1_g010.1.mk* | |  | | phenylalanine ammonia-lyase | |
| ***PtDFR*** | AJO67969.1 | *Pav_sc0002208.1_g840.1.mk* | | Liu et al., 2013; Starkevič *et al*., 2015; | | dihydroflavonol 4-reductase | |
| ***PtANS*** | XP_021803569.1 AEO79983.1 | *Pav_sc0000107.1_g100.1.mk* | | Liu *et al*., 2013; Yang *et al*., 2021a; | | anthocyanidin synthase | |
| ***PtUFGT*** | AEO79979.1 | *Pav_sc0000138.1_g030.1.mk* | | Liu *et al*., 2013; Starkevič *et al*., 2015; | | UDP glucose: flavonoid 3-*O*-glucosyl transferase | |
| ***PtGST11*** | XP_021823852.1 | *Pav_sc0001124.1_g450.1.mk* | |  | | glutathione S-transferase | |
| ***PtABC10*** | XP_021823084.1 | *Pav_sc0000067.1_g880.1.mk* | |  | | ABC transporter | |
| ***PtPOD1*** | XP_021807322.1 | *Pav_sc0000216.1_g580.1.mk* | |  | | peroxidase | |
| ***PtPOD16*** | XP_021815845.1 | *Pav_sc0000624.1_g2540.1.mk* | |  | | peroxidase | |
| ***PtPOD73*** | XP_021832934.1 | *Pav_sc0002544.1_g080.1.mk* | |  | | peroxidase | |
